# Qualitative Insights From Public Interviews in the United Kingdom on the Acceptability of Risk‐Based Eligibility Criteria for Lung Cancer Screening

**DOI:** 10.1111/hex.70937

**Published:** 2026-11-05

**Authors:** Evangelos Katsampouris, Gemma Hutton, Suzanne E. Scott, Stephen W. Duffy, Mattias Johansson, Hilary A. Robbins, Samantha L. Quaife

**Affiliations:** ^1^ Centre for Cancer Screening, Prevention and Early Diagnosis, Wolfson Institute of Population Health Queen Mary University of London London UK; ^2^ International Agency for Research on Cancer Lyon France

## Abstract

**Background:**

Targeted lung cancer screening programmes in the United Kingdom use multivariable risk models to identify high‐risk individuals. However, public acceptability of targeting screening by risk using eligibility criteria, such as age and smoking history, remains unclear, including the psychological and behavioural consequences of eligibility disclosure.

**Aim:**

To explore UK public attitudes toward the acceptability of current risk‐based eligibility criteria for lung cancer screening and the anticipated psychological and behavioural responses to eligibility disclosure.

**Methods:**

A qualitative study was conducted as the second phase of a sequential explanatory design. Semi‐structured telephone interviews were held with 28 UK adults aged 45–77 years between January–April 2024. Participants were purposively sampled for diversity in demographics, smoking history, and prior acceptability questionnaire responses. Interviews were analysed using applied thematic analysis informed by the Theoretical Framework of Acceptability.

**Results:**

Criteria were generally acceptable when perceived as evidence‐based and logical, particularly age and smoking history. However, participants expected flexibility, suggested expanding inclusion criteria and had concerns about fairness and discrimination especially regarding ethnicity and education. Eligibility was anticipated to motivate screening attendance but also cause worry; the prospect of ineligibility often led to feelings of exclusion, anxiety, and disappointment. Participants emphasised the need for clear, evidence‐based communication of the rationale and individual risk, alongside alternative options or support for those who are ineligible.

**Conclusions:**

Clear and transparent communication of the rationales underpinning eligibility criteria, provided by the screening programme and trusted health professionals, are essential for improving public acceptability and individual understanding of eligibility status. These strategies should also mitigate against potential stigma, perceived discrimination, and over‐reassurance, while ensuring appropriate safety‐netting and risk management for those who are ineligible. Tailored messaging and pathways may optimise engagement, trust, and psychological wellbeing within lung cancer screening programmes.

**Patient or Public Contribution:**

The research team sought the views of eight public and patient representatives with lung cancer experience (either personally or being a carer) during study conception. They endorsed the research objectives, helped to develop participant‐facing interview questions and gave feedback on the appropriateness of the design, agreeing that in‐depth interviews would best capture perceptions of the acceptability of risk‐based eligibility criteria.

## 1 Introduction

Lung cancer remains the primary cause of cancer‐related deaths globally [1]. Screening with low‐dose computed tomography (LDCT) significantly reduces mortality among high‐risk individuals [2, 3], where the benefit‐risk ratio is most favourable [4]. Many countries have introduced targeted lung cancer screening programmes, with eligibility largely driven by age and tobacco smoking history but approaches to risk‐based selection vary. In the United States (US), eligibility requires meeting categorical thresholds: age (50–80 years), ≥ 20 pack‐years smoking history, and smoking within the past 15 years [5].

In the United Kingdom (UK), national roll‐out of lung cancer screening was recommended in 2022 [6], with the national English programme aiming for full coverage by 2030. Individuals are invited to an initial telephone‐based lung health check to assess their lung cancer risk if they are aged between 55 and 74 and have ever been recorded as smoking by their primary care practice. Eligibility for low‐dose CT lung cancer screening is determined using multivariable risk models [7, 8, 9] evidenced to be more sensitive and efficient at identifying who to screen than categorical criteria [10, 11]. These models (Prostate, Lung, Colorectal, and Ovarian Cancer Screening Trial Model 2012; Liverpool Lung Project‐v2^8–10^) calculate individual risk scores using age, tobacco smoking, and additional factors such as personal/family cancer history, respiratory disease, environmental/occupational exposures, race/ethnicity, education level (as an individual‐level proxy measure of socioeconomic group) and body mass index (BMI). Each risk factor contributes to the score according to its coefficient, with smoking history weighted more heavily than others. Those who meet or exceed a specified threshold are eligible to be invited for low‐dose CT screening.

While evidence supports the clinical utility of risk prediction models for identifying those most likely to benefit from screening [9, 12, 13], no research has examined the public acceptability of restricting screening to high‐risk individuals based on the specific risk factors used to determine eligibility. Acceptability is a multifaceted concept reflecting how individuals delivering or receiving a healthcare intervention perceive its appropriateness, based on their anticipated or experienced cognitive and emotional reactions [14]. The Theoretical Framework of Acceptability (TFA) [14, 15, 16, 17] defines constructs for measuring this, including coherence (understanding of risk‐based criteria), affective attitude (feelings toward criteria) and ethicality (alignment with individual values).

Little is known about public perceptions of using smoking, a behavioural risk factor associated with stigma, as a criteria to determine eligibility for lung cancer screening [18]. Nor is there significant research exploring public perceptions of using education and ethnicity as eligibility criteria which, in the context of the systemic marginalisation associated with these characteristics, may be challenging to public acceptability [18] and lower screening participation [19]. Evidence is also limited on psychosocial and behavioural consequences of communicating eligibility (or ineligibility), which is in itself a risk communication intervention as eligibility indicates higher risk, but ineligibility indicates relatively lower, rather than no risk. One study found that individuals deemed ineligible for screening during a Lung Health Check appointment experienced higher psychological distress than those eligible, persisting for three months [20].

Furthermore, while ineligibility for lung cancer screening indicates relatively lower risk, risk is neither negligible nor static. Eligibility can change with age or smoking duration, while those who stop smoking may underestimate their risk, particularly if this means they are no longer eligible for screening [21, 22]. Current criteria may also miss many eventual lung cancer cases; fewer than half of UK lung cancer patients meet eligibility requirements [23], and in Canada over a third (37.6%) of patients would have been ineligible due to quitting smoking more than 15 years prior, disproportionately affecting those from Black ethnic backgrounds [24, 25].

Evidence on the public acceptability of risk‐based eligibility from other screening contexts is mixed. A UK community jury study found risk stratification acceptable, if applied fairly and communicated clearly to low‐risk individuals [26]. However, risk‐stratified approaches to determining screening frequency or intervals in breast and cervical cancer have raised concerns [27, 28, 29, 30, 31], with low acceptability of risk‐based criteria implicated qualitatively in non‐participation among Pakistani women [32].

This is the first study to explore the UK public's attitudes toward the acceptability of specific risk‐based criteria used to select individuals for a national lung cancer screening programme. We explored factors affecting perceptions of acceptability among adults approaching or of screening‐eligible age, and their anticipated psychological and behavioural responses to eligibility disclosure. The goal was to generate evidence to inform population‐ and individual‐level communication strategies that increase public understanding and acceptability of risk‐based eligibility criteria and optimise individuals' psychological and behavioural responses to receiving both eligibility and ineligibility statuses.

## 2 Methods

### 2.1 Study Design

This qualitative study was the second phase of a two‐phase mixed‐methods design. The first phase of the study was a cross‐sectional online survey with UK adults voluntarily recruited through a research agency with a large panel of members (Dynata Global UK Ltd). Participants could express their interest in taking part in the qualitative study at the end of the survey. The qualitative study aimed to provide in‐depth exploration of participants' beliefs and perspectives regarding the risk‐based eligibility criteria for lung cancer screening to complement, extend and explain the findings of the quantitative survey. The qualitative study used remote semi‐structured interviews with English‐speaking UK residents, aged 45–80 years, recruited voluntarily from the first phase online survey. Interviews were completed between January–April 2024. The study is reported according to COREQ guidelines for qualitative research (see Supporting Information: File 1 for completed COREQ checklist).

### 2.2 Participants and Sampling

The sampling frame comprised respondents to a parallel online survey study who were recruited via a research agency (Dynata Global UK Ltd) with a large member panel, using non‐probability sampling. As part of the survey, participants were presented with simple descriptions of the national lung cancer screening programme including the process and benefits of lung cancer screening, and the details and (where considered necessary) explanations for each risk‐based eligibility criterion (e.g., education, ethnicity). The online survey also collected demographic and smoking‐related characteristics, lung cancer risk factors and perceptions, and views towards the acceptability of risk eligibility criteria. On completion of the online questionnaire, participants were asked for permission to be contacted for an interview. Interested participants were emailed an information sheet providing further details of the interview study including the purpose of the study, what was involved and how their data would be handled, as well as a consent form. Participants' responses to the survey informed purposive sampling to select a diverse interview sample using a matrix based on ethnicity, gender, age, area‐level deprivation (Index of Multiple Deprivation (IMD) rank), smoking group, and views on the acceptability of risk‐stratified &QJ11;screening. Purposive sampling by key demographic, smoking and attitudinal variables helped to ensure that interviews were conducted with a range of individuals.

### 2.3 Procedure

Individuals were contacted by telephone by the researcher (EK) who explained the purpose of the research and introduced himself as a researcher working in lung cancer screening. Once eligibility was confirmed, eligible participants were asked to provide further demographic and smoking information (level of education, smoking status), and to give their verbal informed consent for participation, which was audio‐recorded. One‐to‐one, in‐depth semi‐structured interviews were conducted remotely by telephone. The interviews were conducted by EK, a male &QJ0;post‐doctoral researcher with extensive experience in qualitative interviewing and cancer screening research. Participants were offered a £25 shopping voucher as a thank you for their time.

The interview schedule (Supporting Information: File 2) was developed in consultation with public and patient representatives and psychologists, informed by research on the acceptability of risk stratification in cervical screening [33, 34] and the components of the TFA [14]. The interview schedule was used flexibly to cover key topics while allowing unprompted topics to be raised and prioritised by participants and also included brief explanations about the roll out of lung cancer screening in the UK as well as each of the risk‐based criteria. Open‐ended questions focused on views toward the use of specific risk‐based eligibility criteria to decide who is offered lung cancer screening, factors affecting perceived acceptability of criteria, and anticipated reactions to eligibility disclosure.

### 2.4 Public and Patient Involvement

The research team sought the views of eight public and patient representatives with lung cancer experience (either personally affected or being a carer) during study conception. They endorsed the research objectives, helped to develop relevant participant‐facing interview questions and gave feedback on the appropriateness of the design, agreeing that in‐depth interviews would best capture perceptions of the acceptability of risk‐based eligibility criteria.

### 2.5 Analysis

Applied thematic analysis [35] was used to interpret themes relevant to participants' views on the acceptability and anticipated implications of the risk‐based eligibility criteria. Inductive coding was used to ensure participant‐driven code development supported by NVivo (QSR International, v.12/2020). Themes were interpreted and discussed within the context of the Theoretical Framework of Acceptability. The theme hierarchy, including broad themes, subthemes and major codes, is reported in Supporting Information: File 3.

Interviews were transcribed by a professional transcription company and checked by EK for accuracy. Transcripts were not returned to participants for verification to reduce participant burden. Data familiarisation began during the interviews and continued through repeated transcript readings. EK initially coded all transcripts inductively to identify participant derived codes. Coding was then discussed with SS, before developing a codebook based on the primary analytical aims and broader participant‐derived codes and then applied to the transcripts. The codebook (i.e. broader codes) was used by the research team (EK, GH and SQ) to organise the results into major and minor themes, and to identify any fit with the components of the TFA. Disagreements were resolved through multiple discussions and reflection until consensus was reached.

## 3 Results

Of 4876 individuals who completed the online questionnaire, 3093 (63.4%) agreed to be contacted to take part in an interview. One hundred and twenty were initially invited to take part in the interview study and 90 (75% of 120 invited) expressed their interest to participate. Of the first 33 who were identified as eligible, 28 (84.9%) individuals (Mean age = 57 years, SD = 8, range: 45–77) (14 females) participated (Mean duration of interview = 35 min, SD = 5, range: 25–47) (Table 1). The remaining participants were unable to be contacted. A sample of 28 was considered appropriate as it aligns with current qualitative research practice providing sufficiently rich and varied accounts to support an in‐depth thematic analysis and achieve sufficient information power. Consistent with the five dimensions outlined in Malterud, Siersma and Guassora's information power approach [36], the sample size of 28 provided sufficient information power due to (1) highly focused study aims (2), purposive sampling to ensure diversity of perspectives (3), utilisation of an established theoretical framework, the Theoretical Framework of Acceptability, to guide data collection and analysis (4), detailed, rich interview content and (5) in‐depth focused thematic analysis.

**Table 1:** Participants' demographic and smoking characteristics.

	*N*
Gender	
Male	14
Female	14
Highest level of education	
Finished school at or before the age of 15	1
CSE/GCSE/O'level/ONC/BTEC or equivalent	8
A‐levels or equivalent	6
Higher education qualification below degree	1
Degree or higher degree	12
Ethnicity	
White	15
Mixed/Multiple	1
Asian	4
Black	7
Other	1
Smoking status	
Currently smoke	10
Formerly smoked	13
Never smoked	5
Index of Multiple Deprivation (IMD) rank quintile	
Quintile 1 (most deprived)	6
Quintile 2	10
Quintile 3	5
Quintile 4	4
Quintile 5 (least deprived)	3

Themes were organised into three categories (1): *How acceptable are the risk‐based eligibility criteria for lung* screening? (2) *What does it mean to be eligible or ineligible for lung screening?* and (3) *Recommendations for communication about the risk‐based criteria.* Theme descriptions are supported by illustrative quotes (including participants' gender: (M) Male; (F) Female, ethnicity: (W) White; (M) Mixed/Multiple; (A) Asian; (B) Black; (O) Other, smoking group: (C) Current; (F) Former; (N) Never) with further evidence in Supporting Information: File 4. Detailed comparative analysis by smoking status was not undertaken, as the study aimed to generate in‐depth insights into how participants interpreted the eligibility criteria and formed views on acceptability. Associations between smoking status and acceptability were examined in the parallel survey. A brief post‐hoc exploratory analysis of the qualitative data found no consistent differences in views expressed according to smoking status, however, these findings are purely descriptive and based on small numbers of participants who have never smoked (*N* = 5).

### 3.1 How Acceptable Are the Risk‐Based Eligibility Criteria?

Four themes centred around acceptability of the risk‐based eligibility criteria and describe the nature, explanatory factors and conditions that shaped perceptions of acceptability.

#### 3.1.1 Risk‐Based Eligibility Criteria Were Acceptable When Seemingly Logical, Familiar or Based on Evidence

Participants often initially reflected that screening should “*be open to everybody*”*(M/W/FS)* as “*nobody can say they are exempt from the possibility of cancer*”*(M/W/F)* with participants highlighting that they would “*rather have something [screening] before you catch something*”*(M/O/FS)*. However, on further discussion, most participants recognised the need to use available evidence to target screening towards at‐risk populations with an awareness that “*in an ideal world, everybody should be invited but in the real world we have to make decisions*” *(F/O/FS)*. This awareness appeared to be rooted in their assumption that this was necessary to balance limited NHS resources with screening benefits and that “*it's sensible to edit the amount of people that you can target and go with where the chances of people contracting lung cancer are highest*”*(F/W/FS)*.

Regarding individual criteria, most participants accepted smoking history as a basis for screening allocation, citing their understanding of the evidence linking smoking and lung cancer and that “*those people that have smoked will be in the higher risk group*” *(F/O/FS)*. Similarly, age‐based eligibility was viewed as a fair and well understood way to prioritise those at higher risk that was consistent with other screening programmes where “*once you reach a set age then you are called for screening and…that's how it should be [with] lungs*”*(F/W/FS)*. Eligibility based upon personal, or family lung cancer history was also described as acceptable with participants perceiving these to be logical reasons to determine eligibility due to their awareness that “*there are clear links genetically when someone has particular genes and they run in the family then you can be more susceptible to a disease*”*(F/W/FS)*.

Views on ethnicity and education were more mixed. Some participants accepted their use highlighting that “*if there's a certain ethnic minority group that's more likely [to get lung cancer] then maybe offering them screening or lowering the age and offering them screening more…. makes sense*” *(F/O/CS)* and perceiving that “*if it is that there is a much higher risk as in that type of population is at more higher risk, then I think yes they should be included in screening*” *(F/W/FS)*.

Across all the criteria, participants emphasised that understanding the criteria as evidence‐based was central to their acceptability, highlighting that “*you have to look at the &QJ0;evidence, the data*” *(F/O/CS)*. Participants' acceptance of specific criterion was therefore closely tied either to their awareness of this evidence or their implicit trust that such evidence must exist for each criterion to have been included and that it “*makes sense if they can identify a link with lung cancer and people, which they must do if they're using that as a criteria*”*(F/O/FS)*.

#### 3.1.2 Challenges to Acceptability: Requiring Flexibility and Expanding Inclusion

Participants' accounts highlighted several aspects of the criteria that were less acceptable, as well as conditions for acceptability that were not met in practice or that ultimately undermined their initial acceptance. Participants spoke about the need for flexibility when applying the risk eligibility criteria (which are fixed in current practice) so screening could be “*opened up to as many groups as possible who may be potentially vulnerable*” *(M/O/NS)*. This was most apparent for age and smoking‐based criteria, where flexibility was perceived as essential to include those who feel at risk but do not meet current thresholds. This was particularly the case with regard to those who have not smoked but have been exposed to second‐hand smoke with participants highlighting that “*there is evidence of people who had not smoked but had lived around those that smoked, go on to get lung cancer*”*(M/O/CS)* and suggesting that the “*criteria for screening should be extended beyond what it is now*” *(F/O/CS)* to include those exposed to second‐hand smoke.

Regarding age, some felt the lower age limit of 55 is “*counterintuitive*” *(M/O/CS)* because smoking‐related damage occurs at a younger age, making detection too late. This was highlighted as particularly salient for those who started smoking young, had a long smoking history, or met multiple other risk criteria such as having a family history or working in “*dangerous occupations*” where participants felt that people should be “*screened much earlier*”*(F/W/NS)*. Participants also reflected that although it would “*be expensive to bring people in at a lower age*” *(F/W/NS)* there may be reduced costs later due to earlier detection and treatment of lung cancer.

Other participants proposed expanding the inclusion criteria suggesting risk factors they viewed as important or better indicators of risk than current criteria, including medical history (e.g., cardiovascular events, lower immune system) and criteria related to income and living conditions (particularly presence of damp and living near busy polluted roads), which they felt better captured environmental risk than education level.

Participants seemed to automatically assume and expect that because they were aware of evidence linking these exposures to increased lung cancer risk, they would also constitute existing eligibility assessment (which they do not currently).

#### 3.1.3 Criteria as Potentially Unfair, Discriminatory, Irrelevant and Stigmatising Because Lung Cancer Can Affect Anyone

A few participants described the use of eligibility criteria as potentially discriminatory or “*extremely unfair*” *(M/O/NS)*, expressing concern that “*there will always be people who will get lung cancer that don't fall into those groups*” *(F/O/FS)*. This was frequently raised by participants who did not meet the age or smoking criteria who described feeling excluded and suggested that these criteria “*heavily discriminate against the younger patients who have never smoked*” *(*F/O/FS).

Negative perceptions of the smoking criteria also seemed to stem from the perception that individuals who had made, in their view, the choice to smoke would have a greater health advantage through their screening eligibility. Therefore, excluding people who have never smoked was perceived as unfair, with one participant suggesting people who smoke should be put “*at the back of the queue*” *(M/W/FS)*. Meanwhile others expressed concern about precisely these views, warning how “*rationing*” using smoking criteria could be misinterpreted as blaming individuals for their smoking because “*by asking questions about lifestyle choices you are assigning blame*” *(F/O/FS)* which may result in individuals “*keeping it at arm's length rather than embracing it [lung screening]*” *(M/W/FS)*.

Excluding those without a family history was also seen as unfair by some participants, because anyone can develop lung cancer. Participants were often in two minds here. For example, one participant *(F/O/FS)* described that on the one hand it “*makes sense obviously [and is] fair*” to use personal and family lung history as criteria, but at the same time it is “*discriminatory*” and “*unfair*” against those who would not meet these criteria but could still get lung cancer.

Similarly, concern was expressed about the potential for the ethnicity and education criteria to be discriminatory, with some participants opposing their use on this basis. There was concern that they could lead people to “*feel very, very intimidated*” or “*embarrassed*” *(M/O/FS)*, and would be “*like a stigma*” for those ethnic groups targeted and prioritised by the criteria. Opposition towards using these criteria appeared to be compounded by their perceived irrelevance, with participants suggesting that someone's *“previous history”* is “*more important* [for lung cancer risk*] than ethnicity or your background*” *(F/O/CS)*. Basing eligibility on unchangeable traits like ethnicity also felt particularly unfair to some, leading to feelings of exclusion based on something “*I can't do anything about*” *(F/W/NS)*. Education was viewed as difficult to measure and not necessarily indicative of a particular life trajectory or risk behaviour as “*anyone, whether you've been educated well or not,… can still get cancer*” *(W/F/CS)*. The utility of applying ethnicity and education criteria in addition to smoking history was therefore further questioned, with some participants describing how targeting smoking behaviour would “*indirectly end up targeting certain [higher risk] groups in society*” *(M/O/NS)* anyway. Furthermore, that using education might not improve access, as wealthier and more educated individuals could “*afford to go private*” *(W/F/CS)*, while those at higher risk might still decline screening. Perceiving there to be a justifiable evidence base was highlighted as instrumental to gaining public support for using these criteria and for mitigating the potential for individuals to experience these criteria as discriminating and unfair.

#### 3.1.4 Acceptability Limited by Lack of Public Information About Lung Cancer Aetiology, Incidence and Symptoms

A lack of public information about the aetiology and incidence of lung cancer beyond smoking and personal lung health history appeared to undermine participants perceived acceptability of specific eligibility criteria and this is reflected throughout many of the other themes. Family history, ethnicity and education were least known and understood. While some understood the rationale for including family history, and called for it to be a standalone criterion, others doubted heredity played a role, expressing that they “*didn't think lung cancer is a genetic condition* [but instead]…*a condition caused by either smoking or toxic substances or environmental factors*”*(M/O/NS)*. How ethnicity and education could “*possibly play a part in determining whether or not you get lung cancer* “(F/W/NS) was also questioned, and participants subsequently described low perceived fairness and limited understanding of why these factors were considered relevant to determining eligibility. Misunderstandings extended to the use of education, with one participant concerned that individuals from lower socioeconomic backgrounds would be excluded based on criteria that deem it “*not worth saving that person's life*” *(M/O/FS)*. Additionally, some participants assumed symptoms were relevant, saying they would “*ignore*” *(M/W/CS)* invitations to screening if asymptomatic, thinking themselves unlikely to benefit, while others described how “*anyone who's got [lung cancer] symptoms*” *(*F/O/CS) should be offered screening.

### 3.2 What Does It Mean to Be Eligible or Ineligible for Lung Cancer Screening?

Participants expressed a range of psychological and behavioural responses when asked how they anticipated reacting to being eligible or ineligible for screening and offered insight into how best to communicate eligibility status.

#### 3.2.1 Eligibility as Motivating and Empowering

Most participants indicated that they would “*a hundred percent*”*(F/O/CS)* attend screening if eligible, motivated by the potential to detect lung cancer early when *“something can be done about it”* (F/W/CS) and the psychological reassurance of gaining “*peace of mind*”*(F/W/FS)* about their lung health. While some acknowledged that eligibility implied elevated risk, this concern encouraged them to “*make sure I have the test [screening]*” *(M/W/FS)*. Some participants supported being informed about what eligibility means in terms of elevated lung cancer risk. Participants described how this knowledge would empower them to make informed health decisions, “*look for help*”, and encourage screening attendance. It was important to receive the “*whole picture*” and be “*fully informed*” about why they were eligible and what it meant, rather than “*giving people half information*”*(F/O/CS)*.

#### 3.2.2 Eligibility as a Cause for Concern and Implications for Communication

Some participants expressed that finding out they were eligible would be worrying and “*a bit frightening*” *(M/O/FS)* causing them to hesitate about taking part and fear that screening might reveal “*you're going to hear something you don't want to hear*” *(F/O/FS)*.

There were suggestions to allow individuals to choose “*whether they want to find out or not”(M/W/FS)* about their individual risk information or even to delay any direct discussions about individual risk until after screening or beyond, by “*do[ing] it in stages*” to avoid causing negative psychological reaction or “*panic a lot of people*”. However, some participants emphasised the importance of sharing risk information and that there was no need to “*pussyfoot over it*” *(M/W/NS)* as it may encourage people to “*make sure I have the test*” *(M/W/FS)*. Others believed that knowing they are at eligible and at higher risk “*might put people off having the screening*.” *(M/O/FS)* and would need “*very careful explanation*” *(M/W/NS)*. Some participants suggested that it would be best to follow the pattern of other screening programmes where you are informed of your eligibility due to age and not provided with personalised risk information. Participants recommended that it may be better to “*not go into the ins and outs of it*” but instead “*just get the lette*r *saying that you're eligible. please ring to make an appointment*” *(F/W/CS)*.

Some participants further suggested that communications about eligibility should be framed positively to accentuate the increased chance of benefiting from screening for certain groups or the actual benefits themselves: “*The higher the risk, the more likely the need for screening. Or more importantly the more likely the benefit from screening*” *(M/W/FS)*. There was also suggestion that “*depend[ing] on the person and [their] level of support*”*(M/O/CS)* and “*because some people do not react well to letters*”(*M/O/CS*), it would be better for some people to be told they are eligible by telephone or in a face‐to‐face appointment by a health professional where there would be opportunity for discussion.

#### 3.2.3 Left Out, Disappointed and Worried About Risk Due to Ineligibility; Why Not Me?

Many participants anticipated that they would “*feel excluded*”, “*upset*” and “*annoyed*” if not invited for screening or underwent eligibility assessment “*then at the end was told no*”*(F/W/NS)*. Participants worried that ineligibility could delay lung cancer detection and risk worse outcomes describing how they “t*echnically could die because it wouldn't be picked up*” *(M/W/NS)*. Some linked this to a sense of unfairness, feeling entitled to screening after contributing to the NHS, having “*paid my tax and national insurance*”*(M/O/FS)*, and questioned the cost‐effectiveness of delayed diagnosis as something that would not “*make economic sense… to find out in the next 10 years that the cancer has worsened [because] it will cost a lot to treat it*”*(M/O/CS)*.

Feelings of exclusion and worry seemed particularly common among individuals who felt at heightened risk due to their being a “*heavy smoker*” or risk factors that fell outside of the eligibility criteria, particularly for individuals with histories of second‐hand smoke exposure.

#### 3.2.4 Acceptability, Alternatives and (False) Reassurance If Ineligible

Other participants anticipated they “*wouldn't be aggrieved*” and would be “*happy*” to accept ineligibility, recognising that “*some people will need to be prioritised over others*” *(F/O/FS)*. There was some concern that ineligibility may give individuals *a* “*free pass to start smoking again*” with suggestion that those who are ineligible should be advised that their risk may increase “*if they you start smoking again*” *(F/W/FS)*.

Some reflected that they would feel more accepting if ineligibility seemed temporary (“*maybe they might invite me one day)*”*(M/O/FS*) or if alternative options were available, such as visiting a GP: “*so you're essentially not excluded, you have the option of doing something [visiting GP] yourself*”*(M/W/CS)*. Others reported that they would seek reassurance or ask their GP about “*find[ing] what other available resources are there for people who do not qualify for that screening*” *(M/O/CS)*.

Some interpreted ineligibility as reassuring and expected that they would “*accept it on the basis that I don't have any problems with my lungs*”*(M/O/NS)* and that they would not worry about being ineligible unless they developed symptoms, in which case they would contact their GP. Several participants commented that if they were ineligible for screening, they would wish to know “*which of those criteria excluded me*” *(F/W/NS)* so that they could make sense of and contextualise their ineligibility.

### 3.3 Recommendations for Communication About the Risk Eligibility Criteria

Participants were asked for suggestions about how best to communicate the use of the risk‐based eligibility criteria to the public and made a number of recommendations.

#### 3.3.1 The Importance of a Clear and Evidence‐Based Rationale When Communicating (In)Eligibility

“*Actually explain[ing]*” the criteria to the public was seen as instrumental to support public understanding and acceptability of both the targeted screening approach and individual eligibility outcomes. Participants talked about the importance of simple language and “*communicating messages that are clear, easy to understand… and that patients don't miss out on any important information*” *(F/O/FS)*. Participants emphasised that sharing the evidence underpinning the criteria, as opposed to simply stating the criteria with an expectation of unquestioning public acceptability, was key to providing a clear and convincing rationale.

#### 3.3.2 Variety and Flexibility in the Way the Risk‐Based Eligibility Criteria Are Communicated

Participants repeatedly highlighted the need for a broad mass marketing campaign aimed at increasing awareness and understanding of the criteria and screening programme. Participants suggested that the “*ground would have to be prepared through, you know, a big advertising campaign*” to ensure that individuals were not “*surprised if you get a call*”.

Many participants recommended a varied approach to communication to take into account variations in digital literacy and access to ensure this information is accessible for everyone as “*social media is for certain generations definitely*” whereas “f*or the older generation ….a letter through the post, posters at the GP's, posters at the hospital*” (F/O/CS). Letters from the GP or other official NHS service were often reported as the preferred way to receive information about the risk criteria as recipients were more likely “*to take notice of it*” (F/W/CS), and letters were perceived as more accessible with information that could easily be shared with family members.

## 4 Discussion

This is the first study to seek the UK public perspectives on the acceptability and anticipated psychological impact of lung cancer screening eligibility criteria. Participants in the study were broadly accepting of using risk‐based eligibility criteria when the rationale and evidence‐base were well explained, perceiving this as a necessary approach to prioritise resource for those at high risk, albeit on the assumption that the rationale was solely to optimise limited NHS resources rather than there being an unequal ratio of harm for low‐risk populations. This was particularly the case for risk factors which felt familiar and logical (such as age, smoking and family history) aligning with prior research [37]. However, views were mixed, with participants expressing greater concern about the use of education and ethnicity, particularly regarding the potential for stigma and discrimination. Misperceptions around the need for symptoms to qualify for screening and gaps in knowledge about incidence and risk factors for lung cancer were also identified. Furthermore, acceptability of the eligibility criteria was often conditional on their assumed flexibility and the inclusion of additional risk factors, which the criteria do not allow for in practice. Predominantly positive views were reported towards eligibility with participants also reporting high anticipated engagement with screening if eligible. However, ineligibility was identified as more challenging for some participants. Our findings raise some key challenges that may require addressing to facilitate the successful implementation of the risk‐based eligibility criteria.

Acceptability was predicated on participants' understanding of the evidence underpinning the use of the risk‐based criteria. Combined with the identified gaps in knowledge and understanding of lung cancer risk factors, and deficiencies in public information, these findings highlight the need for clear, transparent and evidence‐based communication to “actually explain” and justify the use of each risk‐based criterion. Such communication was viewed as important for mitigating perceptions of discrimination, stigma or unfairness, echoing findings from other risk assessment research [38]. Participants highlighted the need for a comprehensive public communication strategy utilising multiple media channels to ensure that this evidence is disseminated before individuals receive screening invitations. Large scale awareness and engagement campaigns have been effective for other cancer screening programmes [39]. Public awareness strategies could inform the public about the rationale for risk‐based criteria and address participants' calls for greater transparency regarding the evidence base underpinning their use.

Stigma and discrimination were identified by our participants as potential issues related to the use of smoking, ethnicity and educational level as criteria. Exclusion due to these factors may lead to feelings of discrimination, while inclusion may lead to perceptions of stigma and blame. Dennison et al. argue that “*the potential discrimination inherent in risk‐stratified screening can be justified as long as it is central to the success of such programmes, rather than reflecting arbitrary judgment*s” [40]. This corroborates our participants' accounts, regarding the importance of transparently communicating the evidence‐based rationale for each constituent criterion. Perhaps especially so when criteria concern characteristics for which communities have historically experienced marginalisation, systemic discrimination and institutional racism (i.e., ethnicity and socioeconomic group), potentially perpetuating mistrust of screening and deterring participation [41]. However, Dennison et al. also caution that information alone cannot fully mitigate the stigma associated with smoking‐related eligibility [40]. Stigma is well documented among people who smoke and is linked to blame, guilt, poorer mental health, delayed help‐seeking [42, 43], and lower screening uptake [44]. Several strategies have been proposed to reduce this stigma in lung cancer screening communication including the use of person‐first language (“person who smokes” rather than “smoker”) [45], acknowledging the generational context of tobacco promotion when the eligible cohort began smoking [18] and applying stigma assessment tools such as the Lung Cancer Communications Assessment Tool [46]. Health professionals also play a pivotal role, with communication training and co‐designed resources targeting health professionals' communication strategies around smoking identified as promising approaches to reduce stigma [47, 48]. International differences should be considered as smoking stigma tends to be greater in countries with more restrictive smoking legislation and less socially normative, potentially reducing acceptability of smoking related criteria in these settings [49].

Tackling the stigma and discrimination associated with ethnicity and educational level as risk‐based criteria is less well understood. In the context of our findings that these criteria were less acceptable and evoked more concern about potential stigma and discrimination than smoking as a criterion, further research is critical to inform public‐facing communication strategies that minimise these unintended consequences. In particular, educational level as a criterion was not well understood nor well received by some participants and was also identified as the least accepted criteria in our quantitative survey study of the acceptability of the risk‐based criteria (unpublished/in prep). Although educational stigma remains underexplored, stigma associated with lower educational attainment has been suggested as prevalent and normalised within meritocratic societies contributing to differences in health and social outcomes throughout the lifespan [50]. Asking questions about ethnicity and educational level may evoke distrust and concerns about fairness, potentially undermining the ethicality of the risk‐based criteria and negatively influencing affective responses, key constructs within the TFA, especially amongst groups already less likely to engage with screening [51]. Paradoxically, targeted screening protocols designed to reach high‐risk populations may have the potential to worsen health inequalities by generating feelings of stigma and discrimination, particularly among individuals experiencing intersectional disadvantages relating to ethnicity, smoking status, and lower educational attainment. Co‐designed research to identify acceptable ways of communicating these criteria may therefore be essential. The use of alternative proxies for educational level may help reduce stigma, while the emerging blood‐based risk stratification biomarkers could provide a more acceptable and potentially equitable basis for determining screening eligibility.

Some participants who initially accepted eligibility criteria went on to describe conditions of their acceptability that were at odds with the specific criteria discussed. The most cited issue related to the ineligibility of those who had never smoked but had experienced significant second‐hand smoke exposure. Concern over its absence has been observed previously, alongside tension that anyone can get lung cancer regardless of smoking history [19]. This is understandable in the light of evidence and publicity implicating second‐hand smoke exposure in lung cancer risk [52] as well as the recent publicity around the increasing prevalence of lung cancer in people who have never smoked. Consequently, those participants in the present study who were accepting of current practice, often did so with the condition that the criteria would be revised in the future to include more at‐risk individuals. This highlights the need for clear information and guidance for those who feel at risk but are currently ineligible. Further research is needed to identify the specific information needs of this group and to develop communication guidelines for health professionals, particularly GPs, who are likely to encounter those who are ineligible but perceive themselves to be at risk, particularly those with significant second‐hand smoke exposure. This may be particularly salient in countries where second‐hand smoke exposure is perceived as a significant health risk and viewed less favourably [53].

Ineligibility in general was an aspect of the risk‐based criteria that challenged acceptability among participants with most participants anticipating a negative emotional impact of ineligibility. Increased lung cancer‐specific distress has previously been observed among individuals opting into, but ineligible for, lung cancer screening [20]. Issues around ineligibility parallel research findings on reducing screening frequency for low‐risk individuals in other cancer contexts. While risk‐based screening is generally acceptable, reducing or stopping screening for low‐risk individuals has been found to be far less acceptable than increased screening for high‐risk individuals [54]. Similarly, age‐based discontinuation of screening where individuals have passed the upper age limit for screening has been associated with negative psychological reactions [55]. Recent studies have shown that many would opt for cancer screening even in hypothetical scenarios offering no mortality benefit and potential harm [56, 57]. Scherer et al. attribute this motivation to the psychological gains that screening offers, such as information, reassurance and peace of mind, which are benefits our own participants frequently emphasised [56]. In the context that lung cancer is frequently publicly perceived as one of the most serious cancers [58], alternative strategies for managing risk and anxiety need to be offered in lieu of the reassurance that screening may provide. Indeed, some of our participants reported that they would want clear, individualised explanations for ineligibility and may seek alternative means of accessing screening or reassurance, mirroring findings in breast screening [55]. Wider research has highlighted the importance of health professionals' support and reassurance when implementing risk‐stratified screening, particularly for low‐risk individuals or those facing reduced screening [59, 60]. To address concerns and mitigate anxiety, health professionals should be equipped with communication training and resources to offer appropriate risk management and safety‐netting strategies instead of downplaying risk as a strategy to reassure. Communication should also cover screening harms alongside benefits, explaining how eligibility criteria are designed to protect low‐risk individuals from the potential harms of screening for a very small chance of benefit as well as symptom vigilance. Despite evidence that decision aids can enhance knowledge and understanding of lung cancer screening [61, 62], relatively little attention has been given to the development of information resources for individuals who are ineligible for screening [63]. This gap has begun to be addressed through the recent development of communication resources for ineligible individuals within the Australian National Lung Cancer Screening Program's broader communication toolkit [64]. Our findings highlight the importance of supporting individuals whether eligible or not, and of using the eligibility assessment telephone appointment and emerging digital eligibility tools to proactively provide information tailored to those who are ineligible. In terms of communicating individual risk, participants were divided about whether it would be beneficial to be informed about the increased risk associated with eligibility. Some reported this would be motivating while others felt this may cause anxiety and preferred a generic disclosure approach, like other screening programmes. Previous research demonstrates general acceptability of disclosing a higher risk status, while also identifying subgroups who experience distress, particularly when risk status is unexpected [28]. Given the mixed preferences, further research could focus on how best to discuss eligibility in terms of increased individual risk and to agree on terminology suitable for population‐level messaging.

A further key finding was the limited awareness of lung cancer risk and incidence, particularly for heredity, ethnicity and education level, among participants. This reduced acceptability and fuelled perceptions of stigma and discrimination. Some participants also misinterpreted screening ineligibility as an indication of good health, posing a risk of over‐reassurance. Prior research reports similar misconceptions about the diagnostic capability of risk assessment in cancer screening [27] and warns ineligible individuals may ignore symptoms or delay help‐seeking [65, 66]. Broader evidence shows limited understanding of risk stratification across cancers [59], causal pathways beyond smoking, and the asymptomatic nature of early‐stage lung cancer [58], especially among people who smoke [67]. Within the TFA, intervention coherence and perceived effectiveness are central to acceptability [68]. Clear communication is therefore essential to explain how lung screening works, why eligibility criteria are used and that the criteria are not diagnostic nor do they signify lung health. Consistent messaging at both the population and individual level is crucial to prevent over‐reassurance, mitigate anxiety, promote risk‐reducing behaviour and safety‐net ineligible individuals who may later develop lung cancer.

There were several limitations to our study. Recruitment via participation in our parallel online survey study limits generalisability of our findings to those without access to the internet or with low digital literacy. The use of internet panels for recruitment also increases risk of selection bias and may reflect a more motivated and health aware sample compared to the general population. Indeed, while over half (57%) of our sample lived in areas categorised within the two most socioeconomically deprived quintiles nationally, a substantial proportion (41%) were educated to degree level, which differs from general population distribution for this age group [69]. Furthermore, the survey and interviews were completed in English and subsequently our findings cannot be generalised to non‐English speakers. This is a significant limitation in the context of lower lung cancer screening rates amongst ethnic minority populations in the UK [70]. The interviews were hypothetical regarding participants' anticipated reactions to their eligibility status, reducing their reliability, and it is possible that participants presented more positive beliefs due to social desirability bias around early detection of cancer. Furthermore, most participants suggested that they would accept screening, if eligible, which contrasts with the current relatively low uptake of lung cancer screening. Although efforts were made to minimise bias, the interviewer's professional role as a university‐based researcher with substantial research interest in cancer screening may also have shaped participant responses due to the researcher's positive beliefs around screening and in‐depth understanding of the risk‐based eligibility criteria. Finally, although public and patient representatives informed study conception and the development of the interview schedule, they were not involved in data analysis, which limited the integration of lived experience perspectives in theme interpretation.

## 5 Conclusions and Implications

Risk‐based eligibility criteria for lung cancer screening were broadly acceptable to our participants. However, we identified numerous potential challenges to acceptability that must be considered and addressed to ensure that acceptability facilitates successful implementation. In the context of the TFA components, intervention coherence, ethicality and affective attitude were shown to require particular attention. Critically, the risk of stigma associated with smoking, ethnicity and educational level as eligibility criteria requires careful and transparent explanation and communication to avoid disenfranchising those for whom screening offers the greatest chance of benefit, particularly in countries where stigma associated with these characteristics are more prevalent and normalised. Consideration of the experiences and information needs of ineligible individuals, particularly those who feel at risk due to second‐hand smoke exposure, is vital alongside support and routes for alternative reassurance. Health professionals will have significant responsibility for addressing the issues around stigma and ineligibility, and subsequently the provision of communication training and resources will be paramount. Public health campaigns focused upon building greater public awareness about lung cancer aetiology, the evidence underlying the risk‐based eligibility criteria and the differential chance of benefit from screening for different groups is essential to increase public understanding, reduce misperception and false reassurance, as well as providing a foundation to minimise feelings of stigma and discrimination. Future research must focus upon developing optimal communication strategies for both public health campaigns &QJ0;and health professional interactions that facilitate awareness and provide support for those who may feel excluded or stigmatised by the risk stratification methods.

## Author Contributions

**Evangelos Katsampouris:** conceptualization, methodology, data curation, formal analysis, project administration, writing – original draft, writing – review and editing, investigation. **Gemma Hutton:** formal analysis, writing – original draft, writing – review and editing. **Suzanne E. Scott:** conceptualization, methodology, supervision, formal analysis, writing – review and editing. **Stephen W. Duffy:** writing – review and editing, conceptualization, funding acquisition. **Mattias Johansson:** conceptualization, writing – review and editing. **Hilary A. Robbins:** writing – review and editing, conceptualization. **Samantha L. Quaife:** conceptualization, funding acquisition, methodology, supervision, writing – review and editing.

## Disclosure

Where authors are identified as personnel of the International Agency for Research on Cancer/World Health Organization, the authors alone are responsible for the views expressed in this article and they do not necessarily represent the decisions, policy, or views of the International Agency for Research on Cancer/World Health Organization.

## Ethics Statement

Ethical approval was granted by the Queen Mary Ethics of Research Committee (QMERC23.131).

## Consent

Audio recorded verbal informed consent was obtained from all participants.

## Conflicts of Interest

HR and MJ are named inventors on a planned provisional patent application for the INTEGRAL‐Risk model, a biomarker‐based risk assessment tool for lung cancer screening eligibility. They will not personally receive any royalties or other compensation related to this potential patent. All other authors have no conflicts to disclose.

## Supporting information


Supporting File 1

Supporting File 2

Supporting File 3

Supporting File 4

## Data Availability

The data that support the findings of this study are available on request from the corresponding author. The data are not publicly available due to privacy or ethical restrictions.
