# Supplementary material for: Qualitative Insights From Public Interviews in the United Kingdom on the Acceptability of Risk‐Based Eligibility Criteria for Lung Cancer Screening
Source: Health Expect. 2026 Nov 5;29(6):e70937. doi: 10.1111/hex.70937 (PMC13735213; doi:10.1111/hex.70937)
Supplement: Supplementary file 1 [file HEX-29-e70937-s001.docx]

**Supplementary file 4.**

**Table 1.** Illustrative quotes for each theme descriptions

Basic participant characteristics accompany each quote (Gender: Male;Female, ethnicity: White;Mixed/Multiple;Asian;Black;Other, smoking group: Current;Former;Never)

| 1. **How acceptable are the risk-based criteria for lung screening?** | |
| --- | --- |
| **Risk-based eligibility criteria were acceptable when seemingly logical, familiar or based on evidence** | *“And if the facts and figures show that they are the most susceptible to or at higher risk to, then it makes perfect sense to me.” (F/W/FS)*  *“I think that's the way to go because they can't screen everyone, it'd be too much. Obviously, smoking does cause issues with your lungs and you've got more risk of catching lung cancer by smoking. So yes, it's wise to offer it to smokers and probably elderly people before.” (F/W/CS)*  *“I was thinking about people working with asbestos, that is a good reason for them to go and be screened. As I said, not only who smoke but people who are exposed to anything that can affect their lungs…People should get screened for that, yes. Like mining, anything like that.” (F/O/CS)*  *“That’s very logical, you know, there are clear links aren’t there genetically when someone has particular genes and they run in the family then you can be more susceptible to a disease.” (F/W/FS)*    *“They are more likely to smoke than those who are more educated, because those who are more educated tend to know about the harm of going into that. Therefore, using all those criteria are very important, because it is only by using these criteria that you are able to pinpoint those who are more at risk than others.” (M/O/CS)*    *“I don’t have a problem with using people’s ethnicity, because ethnicity does make a big difference on what people sometimes, illnesses they get and which illnesses they don’t get…I do realise that certain people get certain illness and it depends on their ethnicity, their age and whatever, so if this was screened by ethnicity that’s okay with myself…Because different ethnicities have different problems with their bodies as they get older.” (F/O/CS)* |
| **Challenges to acceptability: Increasing flexibility and expanding inclusion** | *“I think there should be flexibility…I think there does need to be more flexibility. I think I said at the beginning, you know, I think it needs to be opened up to as many groups as possible who may be potentially vulnerable. So, the criteria shouldn’t be limited by age, or, for example, there does need to be some flexibility.” (M/O/NS)*  *“What about people like me who were affected by passive smoking? You know, somebody who lives in a family like I used to live, you know, the smoke was terrible. You know, wouldn’t they be affected as well, why aren’t they included?” (M/W/NS)*  *“Therefore, if you target only smokers, leaving people like me who are secondary smokers, then the whole point is pointless…because even if you don't smoke, you can still have a kind of a second-hand effect of smokers. If I go to my friend’s house and they smoke marijuana or cigarettes or vape, and then I inhale it, therefore I am also in danger, as they are also in danger. Therefore for me, for the NHS or the government, the best thing to do would be to extend this screening to everybody.” (M/O/CS)*    *“To be very honest with you that doesn't make sense at all…We do know that smokers begin very young. From the age of 16, 17, they have already begun and the damage has been caused over a period of time. Therefore, if they are going to wait until they are 50, you might as well forget it because the damage is already done…Therefore, for me that criteria of age will be very counterintuitive. It won't make sense.” (M/O/CS)*    *“I think that’s a little unfair because I did smoke for a number of years, that’s what I am saying I think about the time. I did smoke from when I was fourteen until I was about forty-six, so you are talking about thirty-two years but as, yet I wouldn’t qualify for it because of the age criteria.” (F/W/FS)*    *“Oh yes, I definitely think the age range should be flexible, but if it’s one out of the three, or two out of the three and the only thing that’s stopping me is my age, I think it should be flexible, definitely.” (F/O/CS)*    *“Well, if there’s a family history the age, the 55 age, I think those people should be screened earlier because if there’s a family history, as you say, pneumonia or TB or asthma, these things affect you from an early age.  So, probably they should be screened earlier than 55” (F/W/NS)*  *“I feel like it’s- I don’t know, if someone’s immune system’s low in a sense like- Had cancer somewhere else or, I don’t know, heart, stroke problems, you know, generally health-wise if they’re not well the immune’s lower, they’re not strong enough, something like that.” (F/O/CS)* |
| **Criteria as unfair, discriminatory, irrelevant and stigmatising because lung cancer can affect anyone** | *“I feel excluded to be honest, because of, I’m going to call them pen-pushers, up above have decided I have not ticked the right box, I technically could die because it wouldn’t be picked up. You know, just say, for instance, I had lung cancer now, but I’m not old enough to screen, but next year I become old enough and I’m screened and they detect it, they say oh, you must have had it for a couple of years, those couple of years there could have been great interventions with medical devices and it could save me. But, now I’m going to die.  So, no, I feel excluded, it’s not right.” (M/W/NS)*    *“I feel it’s not fair to me to be excluded because I am not a smoker or never have been a smoker.  It’s not fair that my immediate family would all be excluded for the same reasons. I feel it is unfair. You do feel excluded.  You feel left out” (F/W/NS)*    *“I think this criteria heavily discriminates against the younger patients who have never smoked and nonetheless may have lung cancer because there have been cases.”*    *“I mean it does make sense obviously it is fair for the people invited. But as I said, at the risk of repeating myself, every time you discriminate against a group of people, you are being unfair towards the people you are discriminating against. For example, people who do not have a proper family medical history of cancer but they may still have cancer.” (F/O/FS)*    *“Because I don’t think cancer decides – Anyone, whether you’ve been educated well or not, you can still get cancer…I don't think it should make a difference what your education was like, whether you get screening or not. It doesn't really seem fair, does it?” (W/F/CS)*    *“I don’t think anybody should be excluded for ethnicity, socioeconomic factors or their education.” (M/W/NS).*    *“Why have you [linked] this disease to a certain group? Why don’t you make it generally? So when you say, “Oh, if you are a Black man, you are so-and-so age, there is a possibility for you to get cancer,” so the Black people, they feel very, very bad. Why can’t you make it general? So that is what I believe. That’s it.” (M/O/FS)*    *“So in principle I would probably want to see it targeted more at the have-nots in society if you like, rather than the haves. But my fear is that time and time again the have-nots don’t feel they have got sufficient stake in society to respond to these government initiatives.”  (M/W/FS)*    *“I believe that some patients, especially those who smoke and make lifestyle choices that most people know are not beneficial to health may think that by discriminating against them or by asking questions about lifestyle choices you are assigning blame. Like for example saying he smokes, it was his fault”  (F/O/FS)* |
| **Acceptability limited by lack of public information about lung cancer aetiology, incidence and symptoms** | *“I’m not aware that it’s a hereditary thing, that if it’s in your family, I understand partly the reason, blood pressure and all that. If your parents have diabetes, if your parents have it, you’ve got a chance of catching it. But with cancer, I’m not aware whether it’s genetic make-up or not.” (M/O/FS)*    *“I am not really quite sure about. I know some things are hereditary I don’t know if lung cancer is hereditary or if it is an aspect that’s part of lifestyle, smoking, work whatever you have had the high-risk job.” (F/W/CS)*    *“I don’t know, I’ve not seen any research, but I don’t see how white British or being Asian or being Caribbean makes any difference as to whether you get lung cancer or not.” (F/W/NS)*    *“just because people have lower income or are from a lower area doesn’t necessarily to me, in my eyes, mean that they’ve got more chance of actually getting lung cancer than anyone else.” (F/O/CS)*    *“if I hadn't got the symptoms and I got the letter I would just read it and put it in the bin. But there's going to be one time, maybe it's once or twice a year, and I’ll think, oh, I've got a bit of breathless or whatever the symptoms are, I would respond.” (M/W/CS)* |
| 2. **What does it mean to be eligible or ineligible for lung screening?** | |
| **Eligibility as motivating and empowering** | *“I agree with it and wholeheartedly – and if I was given the opportunity, I would take the lung cancer screening.” (M/O/FS)*    *“I would say full disclosure is always best. But obviously that's going to stress some people if they have a high risk. Having said that, if you're telling me I'm high risk, A, I'm concerned, B, more importantly I'm going to make sure I have the test.” (M/W/FS)*  *“Yes, I think you should be informed, because then it gives patients the choice of whether they want to go on for further screening or not.” (F/O/FS)*  *“…telling them about the risk or their own particular risk would be a factor to make them go and look for help.” (M/O/CS)* |
| **Eligibility as a cause for concern, and has implications for communicating eligibility** | *“I think it’s an understandable concern that people will get concerned.” (M/W/FS)*    *“…that’s up to them whether they want to find out or not. I don’t think it should change the way they feel. I mean, yes, it’s frightening…it would be stressful…” (M/W/FS)*    *“I think it would frighten them, definitely, until you get the results there’s no need to say anything” (F/O/CS)*    *I don't think you should say, “Because you are at risk of…” because when I get a letter, for example, for a smear test, I don't get the letter saying, “Come in for a smear test because you might get cervical cancer.” It just says, “Come for a smear test,” and I go” (F/O/FS)*    *“Well, if you’re going to give any kind of information it should be given in general terms…certain groups of people in society who have smoked, are potentially prone to lung cancer…I don’t think people are ignorant about [smoking], but I don’t think the level of risk should be very specific to an individual…Just outlining risks to certain groups of people in society.” (M/O/NS)* |
| **Left out, disappointed and worried about risk due to ineligibility; why not me?** | *“I suppose I would be upset that, not upset, but I think, well, why didn’t I get invited for that screening just to see that everything was all right?” (F/W/FS)*    *“I feel excluded to be honest, because of…[they] have decided I have not ticked the right box ... So, no, I feel excluded, it’s not right.”(M/W/NS)*    *“But I think, now, when I’m a heavy smoker, I would like to have screening. So, I would be a bit upset.” (F/W/CS)*  *“if they’ve got it 50 to 74 and then if they’re trying to filter it outside those parameters, I wouldn’t mind being asked age, ethnicity and profession, I wouldn’t mind. I wouldn’t object to it. But, if I was then excluded, I would want to know which of those criteria excluded me.” (F/W/NS)* |
| **Acceptability, alternatives and (false) reassurance** | *“How would I feel? I would understand if I didn’t fill the criteria that, say, the NHS- The criteria they’ve got, I would understand because maybe there are more people that have got more chances of catching lung cancer than me, even though I’m a smoker.” (F/O/CS)*    *“I would make an appointment with the doctor just to find out and that would be for my own peace of mind. I would make an appointment with the doctor and ask why I hadn’t been invited for this screening....I think if I thought there was something wrong, I would go private for something like that. But only if I was refused the screening.”*  *(F/W/FS)*    “*I’d probably just accept it. I would accept it on the basis that I don’t have any problems with my lungs.And me having my lungs tested just for peace of mind. If I had a problem, I would go to my doctor first” (M/O/NS)*    *“Well, I suppose I’d like to know why but if I was excluded, I would obviously think, well, they mustn’t think there’s anything wrong with me. But that’s my logic way of thinking.” (F/W/FS)* |
| 3. **Communication about the risk eligiblity criteira and (in) eligibility** | |
| **The importance of a clear and evidence-based rationale** | *“I think if the criteria’s actually explained to people…it may make them understand the reason why or why not they haven’t been offered the screening…” (F/O/CS)*    *“I think as long as it’s clearly communicated that it’s about the chances and the risk factors then I think most people would understand.” (F/W/FS)*    *“You need to ensure that you are communicating messages that are clear, easy to understand the way, and that patients don’t miss out on any important information.” (F/O/FS)* |
| **Variety and flexibility in the way the criteria are communicated** | *“I think in the first instance there should be some marketing. I know it sounds crazy, but if you put things in the doctor's surgery, not everybody will go to the doctor's surgery. I think if there was either a program or it was on the news…Other than that, you're sending letters or emails to everybody that's registered with the doctor's surgery. Some sort of marketing to start off with and then followed up with a letter from the doctor’s surgery…”(F/O/FS)*  *“We can begin from public places. Posters do play a part. The government has to come up with a massive communication” (M/O/CS)*  *“So are sort of doing it as a generalised information exercise but then sort of personalised when it is your time to be invited for screening.” (F/W/FS)*  *Well I mean you know the ones with criteria probably don’t have phones, don’t have homes and time and time again these are the people that are most at risk. So I think you would have to spread the message through social workers and housing advisors and you know, through the whole network of people that are communicating with those who are economically deprived” (M/W/FS)*  *Well I’m just thinking, I mean I’m on email but someone like my mum, she doesn’t know how to use the internet and that, so letters would be the best.” (F/O/CS)*  *“I think I’d prefer to get a letter through the post ... because if you've got a letter, you take more notice of it, don't you? Rather than just seeing a poster up somewhere. I mean, you could look at a poster in a doctor's surgery and then see it and then just go in and forget about it, you know? But yes, if they send you a letter saying you may be eligible for lung screening, can you contact blah, blah, blah. I’d do that”. (F/W/CS)*  *I think if you are being asked to go for lung screening then I think you should either get a letter or you know, a lot of people now have like the NHS app so you can get a notification on there.*  *“I would prefer to get a letter through the post” (M/W/CS)*  *“I mean, the elderly and that I suppose would need a letter, because I can’t see them using the intervet that much, so. Yes, they would need something written, even for their families to explain to them or something like that. But certainly the elderly would need a letter.” (M/W/FS).* |
