# Supplementary material for: Qualitative Insights From Public Interviews in the United Kingdom on the Acceptability of Risk‐Based Eligibility Criteria for Lung Cancer Screening
Source: Health Expect. 2026 Nov 5;29(6):e70937. doi: 10.1111/hex.70937 (PMC13735213; doi:10.1111/hex.70937)
Supplement: Supplementary file 2 [file HEX-29-e70937-s002.docx]

**Katsampouris et al (2026)**

**Results theme hierarchy**

**Risk based eligibility criteria were acceptable when seemingly logical, familiar or based on evidence**

**Criteria as acceptable when evidence based**

- Acceptability of criteria if supported by evidence
- Criteria to be used to stratify and prioritise people based on evidence

**Criteria as logical**

- Screening for all in an ideal world but there must be compromise
- Makes sense to prioritise what is known to increase risk
- Makes sense as smoking is a known risk factor
- Makes sense as age is a known risk factor

**Familiarity from other screening programmes**

- Use of age
- Screening is not for everyone in other programmes

TFA components: Perceived effectiveness, intervention coherence, ethicality

**Challenges to acceptability: Requiring flexibility and expanding inclusion**

**More flexibility**

- Earlier screening for those at higher risk
- Those with multiple risk factors
- Those with long smoking histories
- Age limit may be too late for some

**Expanding inclusion**

- Offering LCS to second hand smokers
- Screening more people may save money spent on future treatment
- Offer LCS to those with symptoms
- Other criteria suggestions as better indicators of risk (income, living conditions, medical conditions)

TFA components: Affective attitude, intervention coherence, ethicality

**Criteria as potentially unfair, discriminatory, irrelevant and stigmatising because lung cancer can affect anyone**

**Unfair**

- Screening as a right
- Nothing should exclude people from screening
- Why should smokers be at the front of the queue?
- What about second-hand smokers?
- Unfair to use criteria that are static (e.g. education and ethnicity)

**Discriminatory and stigmatising**

- Ethnicity as criteria could be discriminatory
- Education as criteria could be discriminatory
- Targeting groups (education/ethnicity/smoking) could results in stigma and blame
- Reduced screening uptake due to feeling blamed
- **Irrelevant**
- -Ethnicity as irrelevant to lung cancer risk
- -Education as irrelevant to lung cancer risk
- -Higher educated could access screening anyway
- -Targeting smoking indirectly targets certain ethnicities and educational groups

TFA components: affective attitudes, ethicality, intervention coherence

**Acceptability limited by lack of public information about lung cancer aetiology, incidence and symptoms**

Lack of awareness about LC

- about family history as a risk
- about ethnicity as a risk
- about education as a risk

Misunderstanding about LC and screening

- Asymptomatic means does not need screening
- Symptomatic is a reason for screening
- Why should less educated people be excluded?

TFA components: intervention coherence

**What does it mean to be eligible or ineligible for lung screening?**

**Eligibility as motivating and empowering**

**Positive attitude towards attending**

Would attend if offered

- Attend for peace of mind
- Attend so that something can be done
- Attend because offered

**Eligibility as empowering**

- Knowledge is power
- Importance of understanding why they were eligible to empower

**Eligibility as motivating**

- To change behaviour
- To attend screening

**Eligibility as a cause for concern, and its implications for communication**

**Fear and worry**

- Due to possible lung cancer diagnosis
- Understanding that eligibility signifies being high risk

**Need for careful communication**

- Choice over what you are told
- Some people may panic from communication
- Might put people off going to screening

**Debate over how to share risk information**

- Don’t give too much information as it’s frightening
- Follow the format of other screening programmes rather than disclose the risk information
- People deserve to be fully informed of their risk
- Simple communication prior to appointment to prevent worry

**How to communicate eligibility**

- Focus on benefit rather than risk
- Letters might not be enough for some people
- Letters are familiar means of communicating about screening

**Left out, disappointed and worried about risk due to ineligibility: why not me?**

**Ineligibility and feeling excluded and left out**

- Why not me?
- Having risk factors outside of the criteria
- Does not make sense as will cost more to treat people if diagnosed later

**Ineligibility and feeling worried and disappointed**

- Worry about being at risk of worse outcomes
- Worry if have risk factors outside of the criteria
- Screening as a right

**Acceptability, alternatives and (false) reassurance if ineligible**

**Acceptance of ineligibility**

- General acceptance
- Understanding that some people have to be prioritised over others
- Ineligibility might be temporary (e.g. in terms of age).

**Seeking alternatives and reassurance**

- Going to the GP to seek alternatives
- Going to the GP if symptomatic
- Going to the GP for reassurance
- Understanding why they were ineligible

**(False) reassurance**

- Ineligibility as reassuring that lung health is good
- Free pass to smoking

**3. Communication about the risk eligibility criteria**

**The importance of a clear and evidence-based rationale when communicating (in) eligibility**

- Explain the criteria
- Share the evidence for the criteria
- Clear, simple, transparent

**Variety and flexibility in the way the risk-based eligibility criteria are communicated**

- Mass marketing required to increase awareness
- Mass marketing required to prevent surprise
- Use a variety of communication mediums
- To ensure accessible for all (older age/lack of digital literacy)
- Letters as preferred way to share risk criteria (familiar/easy to share)
