# Supplementary material for: Qualitative Insights From Public Interviews in the United Kingdom on the Acceptability of Risk‐Based Eligibility Criteria for Lung Cancer Screening
Source: Health Expect. 2026 Nov 5;29(6):e70937. doi: 10.1111/hex.70937 (PMC13735213; doi:10.1111/hex.70937)
Supplement: Supplementary file 3 [file HEX-29-e70937-s003.docx]

**Supplementary File 1**

**COREQ 32-Item Checklist**

| **Item No.** | **Topic** | **Guide Questions/Description** | **Reported on Page No.** |
| --- | --- | --- | --- |
| **Domain 1: Research Team and Reflexivity** | | | |
| *Personal characteristics* | | | |
| 1 | Interviewer/facilitator | Which author(s) conducted the interviews. | [Page 7, Procedure] |
| 2 | Credentials | What were the researcher’s credentials? | [Page 7, Procedure] |
| 3 | Occupation | What was their occupation at the time of the study? | [Page 7, Procedure] |
| 4 | Gender | Was the researcher male or female? | [Page 7, Procedure] |
| 5 | Experience and training | What experience or training did the researcher have? | [Page 7, Procedure] |
| *Relationship with Participants* | | | |
| 6 | Relationship established | Was a relationship established prior to study commencement? | [None, drawn from survey panel] |
| 7 | Participant knowledge of the interviewer | What did the participants know about the researcher (e.g., personal goals, reasons for doing the research). | [Page 7, Procedure] |
| 8 | Interviewer characteristics | What characteristics were reported about the interviewer? E.g. Bias, assumptions, reasons and interests in the research topic | [Page 29, Discussion] |
| **Domain 2: Study Design** | | | |
| *Theoretical framework* | | | |
| 9 | Methodological orientation and theory | What methodological orientation was stated to underpin the study? E.g. grounded theory, discourse analysis, ethnography, phenomenology, content analysis | [Page 8, Analysis] |
| *Participant selection* | | | |
| 10 | Sampling | How were participants selected? | [Page 6, Participants and Sampling] |
| 11 | Method of approach | How were participants approached? | [Page 6/7, Participants and Sampling/ Procedure] |
| 12 | Sample size | How many participants were in the study? | [Page 10, Results] |
| 13 | Non-participation | How many people refused to participate or dropped out? Reasons? | [Page 10, Results] |
| *Setting* | | | |
| 14 | Setting of data collection | Where was the data collected? | [Page 7, Procedure] |
| 15 | Presence of non-participants | Was anyone else present besides the participants and researcher? | [Page 7, Procedure] |
| 16 | Description of sample | What are the important characteristics of the sample? | [Page 10, Table 1 / Results] |
| *Data collection* | | | |
| 17 | Interview guide | Were questions, prompts, guides provided by the authors? Was it pilot tested? | [Supplementary File 1, Page 7 Methods] |
| 18 | Repeat interviews | Were repeat interviews carried out? | [Page 7, Procedure] |
| 19 | Audio-/visual-recording | Did the research use audio or visual recording to collect the data? | [Page 7, Procedure] |
| 20 | Field notes | Were field notes made during/and or after the interviews? | [No, field notes were not felt necessary] |
| 21 | Duration | What was the duration of the interviews? | [Page 10, Results] |
| 22 | Data saturation | Was data saturation discussed? | [Page 10, Results] |
| 23 | Transcripts returned | Were transcripts returned to participants for comment and/or correction? | [No, to reduce participant burden/retain anonymity] |
| **Domain 3: Analysis and Findings** | | | |
| *Data Analysis* | | | |
| 24 | Number of data coders | How many data coders coded the data? | [Page 8, Analysis] |
| 25 | Description of coding tree | Did authors provide a description of the coding tree? | [Page 8, Analysis, Supplementary File] |
| 26 | Derivation of themes | Were themes identified in advance or derived from the data? | [Page 8, Analysis] |
| 27 | Software | What software, if applicable, was used to manage the data? | [Page 8, Analysis] |
| 28 | Participant checking | Did participants provide feedback on the findings? | [No, to reduce participant burden] |
| *Reporting* | | | |
| 29 | Quotations presented | Were participant quotations presented to illustrate the themes/findings?  Was each quotation identified? | [Page 11-21, Results] |
| 30 | Data/findings consistency | Was there consistency between the data presented and the findings? | [Page 11-21, Results] |
| 31 | Clarity of major themes | Were major themes clearly presented in the findings? | [Page 11-21, Results] |
| 32 | Clarity of minor themes | Is there a description of diverse cases or discussion of minor themes? | [Page 11-21, Results] |

Developed from: Tong A, Sainsbury P, Craig J. Consolidated criteria for reporting qualitative research (COREQ): a 32-item checklist for interviews and focus groups. International Journal for Quality in Health Care. 2007. Volume 19, Number 6: pp. 349 – 357
