# Supplementary material for: Qualitative Insights From Public Interviews in the United Kingdom on the Acceptability of Risk‐Based Eligibility Criteria for Lung Cancer Screening
Source: Health Expect. 2026 Nov 5;29(6):e70937. doi: 10.1111/hex.70937 (PMC13735213; doi:10.1111/hex.70937)
Supplement: Supplementary file 4 [file HEX-29-e70937-s004.docx]

Interview Schedule/Topic Guide

**Exploring Public Acceptability of, and Anticipated Psychological Responses to, Risk-Based Eligibility Criteria in Lung Cancer Screening**

**Participant’s no: __________**

**Interview date: __________**

**Time begun: __________**

**Time ended: __________**

**Interview duration: __________**

**Introduction and Ethics**

- Researcher details
- Before we start, I would like to remind you of your rights as a participant in this study. Everything you discuss today will be kept strictly confidential and anonymous. If you are quoted in any of our presentations, reports or publications, your name will never be included, and you will not be identifiable in any way. Your participation is entirely voluntary. We do not have to talk about anything you feel uncomfortable about, and you are free to decline to answer any questions I will be asking. You can end the interview and withdraw yourself from the study at any time without giving a reason. If there are any questions that you are not comfortable answering I would rather you did not answer than tell me what you think I might want to hear. So, if you would prefer not to answer a question, please simply state it and I will move straight onto the next question. So that I do not need to take notes, I will audio-record our conversation. Would that be, OK?
- Aim: As you have read in the participant information sheet, we are interested in listening to your views, attitudes, thoughts, and perceptions towards the use of different criteria to decide who is invited for lung screening and the factors that might affect how acceptable people find this. This will help us to better understand how best to communicate these criteria to the public if a national lung cancer screening programme is introduced, but also to those who might be invited to have their screening eligibility assessed.
- I have a few questions noted down here of things I would like to ask you, but I would really like you to do most of the talking. I want to hear your views so please feel free to be as frank and open as you wish. There are no right or wrong answers. At the end of the interview, there will be an opportunity for you to add anything else that you felt was important and not covered in the questions asked. If you have any questions as we go along or if at any time you do not understand what I am asking and need some clarification, please just ask. Do you have any questions before we begin?

*[Interviewer note: The following questions and topics are intended as a rough topic guide and are not definitive. They may be used flexibly or adapted in response to information provided by the participant. Participants will not be asked all questions. It will depend on each participant’s individual responses and time constraints and based on topics participants would like to discuss.]*

**Warm-up question**

1. Have you ever taken part in an interview as part of a research study?

**Existing knowledge/interest in lung cancer screening**

2. Have you ever heard about lung cancer screening?

If yes, what is your understanding about it? Do you know what this is about/anything about this?

Awareness

If no, the interviewer to provide an explanation

**Views towards current lung screening eligibility criteria**

3. Who do you think should be offered lung cancer screening by the NHS?

Universal or certain factors/target screening

Existing perceptions of at risk groups

Thoughts, knowledge, concerns

Understanding and its consequences/implications

Uncertainty, confusion

4. At the moment, the NHS offers lung cancer screening/targeted lung health check in some areas of England and it is hoped that soon lung cancer screening will be rolled out across the UK. People will only be invited if they are aged 55-74 years and either smoke now, or used to in the past, because they are at higher risk of getting lung cancer. Can you talk me through what you think about using age and smoking history to offer lung cancer screening? And why do you think they use these criteria?

Thoughts about why these criteria would be used/perceived relevance

Thoughts, feelings about being excluded – what if they are excluded because they do not satisfy all criteria?

Expectations to be invited/ to be offered LCS Elaborate on smoking criteria

If non-smoker, thoughts, or concerns about (not) being invited

Age range

Other characteristics such as education, ethnicity, IMD

Fair/benefits/risks/acceptability

Passive smoking

Stigma, guilt, self-blame

5. Other reasons that people might be invited for screening include their medical and family history. For example, having had a lung disease now or in the past or having a close relative who was diagnosed with lung cancer. What do you think about offering lung cancer screening to people because of these reasons?

Thoughts about why these criteria would be used/perceived relevance

Thoughts, feelings about being excluded – what if they are excluded because they do not satisfy all criteria?

Expectations to be invited/ to be offered LCS

COPD, bronchitis, asthma, pneumonia, emphysema or tuberculosis

Fair/benefits/risks/acceptability

Other types of cancer

Genetic issues

6. The other reason people might be invited for screening is because they have been exposed to asbestos. What do you think about offering lung cancer screening to people who have been exposed to asbestos, and why?

Fair/benefits/risks

Thoughts about why these criteria would be used/perceived relevance

Thoughts, feelings about being excluded – what if they are excluded because they do not satisfy all criteria?

Expectations to be invited/ to be offered LCS

Fair/benefits/risks

Acceptability

Coal dust, wood dust, or other mineral, rubber, or metal dusts without using protective equipment

Coal mining industry

Pollution

7. There are also some personal characteristics that increase or decrease someone’s chance of being offered lung cancer screening. This is because these are characteristics of groups of people who are more commonly diagnosed with lung cancer. These include sex, ethnicity, and information about the social and economic conditions in which people live. What do you think about using sex, ethnicity and and education – which is a quick way to assess social and economic circumstances to help decide whether someone might be offered lung cancer screening, and why?

Fair/benefits/risks

Thoughts about why these criteria would be used/perceived relevance

Thoughts, feelings about being excluded – what if they are excluded because they do not satisfy all criteria?

Expectations to be invited/ to be offered LCS

Fair/benefits/risks

Acceptability

8. Are there any other factors that you think the NHS should use to invite people for lung cancer screening?

Perceptions of other risk factors for lung cancer

Expectations to be invited/ to be offered LCS

Fair/benefits/risks

Acceptability

9. Why do you think the NHS would only offer lung cancer screening to people at higher risk of lung cancer? Who do you think might be at higher risk?

Ethicality, fairness, acceptability

Risk and benefits

10. Should lung cancer screening be offered to all adults regardless of their age, smoking history or medical history?

Thoughts, feelings about being excluded – what if they are excluded because they do not satisfy all criteria?

Expectations to be invited/ to be offered LCS

Fair/benefits/risks

Acceptability

**Views towards implications of risk-based criteria for personal screening eligibility**

11. Would you want to be offered lung cancer screening?

Why, why not

Benefits/risks

Factors affecting this decision

12. Do you think you would be eligible for lung cancer screening?

Why, why not

Based on what criteria?

Feelings about this (i.e., concerns about thinking oneself eligible or not eligible)

Do they think they should be eligible even if they don’t think based on criteria discussed

13. Would you want to to be asked questions that work out your chance of getting lang cancer in order to find out if you are eligible for lung cancer screening? (note: clarify this is not something the interviewer is offering)

Benefits/risks

Other factors affecting this decision

Thoughts and feelings

Reactions

14. How would you feel like if you were excluded from the national lung screening programme because you did not meet all eligibility criteria?

Thoughts, concerns/worry, emotional consequences - Worry for people not invited or not at risk?

Reactions

Risk perceptions for lung cancer/concern about risk or getting lung cancer, reassurance

Ethicality, fairness, acceptability

Self-referral

How would people perceive the fact that they might not fit into the criteria?

**Views towards future blood test for eligibility**

In the future, it is possible that a blood test might be used to help decide who is offered lung cancer screening. The test would predict somebody’s chance of getting lung cancer in the future. People with a higher chance of getting lung cancer could be offered screening even if they are younger than 55 or have never smoked.

15. What do you think about the NHS using a blood test to help decide who is offered lung cancer screening?

Better than/worse than age, smoking, medical history criteria (should it be used instead or as well)

Acceptability/how does it make them feel/emotions

Fairness/Ethicality

Perceived reasons

16. Would you want to have a blood test to help decide if you are offered lung cancer screening, and why/why not?

Preference for risk assessment vs. blood test

Trust in results/understanding of how the blood test works (and relative to risk assessment)

Potential benefits/risks

Reactions, concerns

What would you encourage/motivate you to do it?

17. How do you think a blood test would work in helping to decide if you are offered lung cancer screening?

Perceptions of accuracy/reliability

What is in the blood

Blood test to replace the risk assessment questions or to be used in addition to/in combination with the risk assessment questions?

**Ways of communicating lung screening eligibility**

18. As we have discussed, not everyone will be offered lung cancer screening. How do you think people should be informed of whether they are eligible or not to have lung cancer screening? How would you like this to be communicated to you/who by?

Communication practices, GP, NHS

Expectations

Insights/recommendations on ways/types of communicating – what might be acceptable

Letters but also other form of communication

Education/awareness about lung cancer

19. When people are told whether they are eligible for lung cancer screening, do you think they should also be told about their level of risk for lung cancer?

How/by who

Risk assessment, GP, healthcare professionals

Explanation desired/required

20. Do you have any suggestions for how the NHS could communicate the reasons why some people are invited, and some people are not?

Key messages/points/explanations that improve/reduce acceptability

Issues to focus on

**Closing questions**

21. What are your thoughts about your lung health now?

Lifestyle

**Close and debrief**

22. Is there anything else you would like to mention that you feel we have not covered?

Thank participant, answer any questions and provide researchers’ contact details. Offer copy of transcript and report. If the participant discloses concerns during the interview suggest they seek help from their GP and/or the Samaritans. Recommend to participant to look at for more information around lung cancer on CRUK and RCLCF.
